# Low-Cycle Fatigue Behavior of Wire and Arc Additively Manufactured Ti-6Al-4V Material

**DOI:** 10.3390/ma16186083

**Published:** 2023-09-05

**Authors:** Sebastian Springer, Martin Leitner, Thomas Gruber, Bernd Oberwinkler, Michael Lasnik, Florian Grün

**Affiliations:** 1 Chair of Mechanical Engineering, Montanuniversität Leoben, 8700 Leoben, Austria; florian.gruen@unileoben.ac.at; 2 Voestalpine BÖHLER Aerospace GmbH & Co KG, 8605 Kapfenberg, Austria; thomas.gruber@voestalpine.com (T.G.); bernd.oberwinkler@voestalpine.com (B.O.); michael.lasnik@voestalpine.com (M.L.); 3 Institute of Structural Durability and Railway Technology, Graz University of Technology, 8010 Graz, Austria; martin.leitner@tugraz.at

**Keywords:** wire arc additive manufacturing, low-cycle fatigue, Ti-6Al-4V, Ramberg–Osgood model, Manson–Coffin–Basquin, cyclic deformation

## Abstract

Additive manufacturing (AM) techniques, such as wire arc additive manufacturing (WAAM), offer unique advantages in producing large, complex structures with reduced lead time and material waste. However, their application in fatigue-critical applications requires a thorough understanding of the material properties and behavior. Due to the layered nature of the manufacturing process, WAAM structures have different microstructures and mechanical properties compared to their substrate counterparts. This study investigated the mechanical behavior and fatigue performance of Ti-6Al-4V fabricated using WAAM compared to the substrate material. Tensile and low-cycle fatigue (LCF) tests were conducted on both materials, and the microstructure was analyzed using optical microscopy and scanning electron microscopy (SEM). The results showed that the WAAM material has a coarser and more heterogeneous grain structure, an increased amount of defects, and lower ultimate tensile strength and smaller elongation at fracture. Furthermore, strain-controlled LCF tests revealed a lower fatigue strength of the WAAM material compared to the substrate, with crack initiation occurring at pores in the specimen rather than microstructural features. Experimental data were used to fit the Ramberg–Osgood model for cyclic deformation behavior and the Manson–Coffin–Basquin model for strain-life curves. The fitted models were subsequently used to compare the two material conditions with other AM processes. In general, the quasi-static properties of WAAM material were found to be lower than those of powder-based processes like selective laser melting or electron beam melting due to smaller cooling rates within the WAAM process. Finally, two simplified estimation models for the strain-life relationship were compared to the experimentally fitted Manson–Coffin–Basquin parameters. The results showed that the simple “universal material law” is applicable and can be used for a quick and simple estimation of the material behavior in cyclic loading conditions. Overall, this study highlights the importance of understanding the mechanical behavior and fatigue performance of WAAM structures compared to their substrate counterparts, as well as the need for further research to improve the understanding of the effects of WAAM process parameters on the mechanical properties and fatigue performance of the fabricated structures.

## 1. Introduction

Additively manufactured titanium alloys are increasingly used for several applications in aerospace structures, see [1]. Especially the manufacturing process parameters and further post-treatments can significantly affect the resulting microstructure and mechanical properties, which are of utmost importance to ensure a safe and reliable design of structural components [2,3,4]. Moreover, a comprehensive evaluation of fatigue strength becomes imperative in scenarios involving cyclic loading. This necessity arises due to the potential influence of process-induced defects, residual stresses, and microstructural conditions on fatigue behavior, especially within the high-cycle fatigue (HCF) regime [5,6]. The HCF regime is delineated by load cycle ranges spanning from $10^{4}$ to $10^{7}$ cycles, as depicted in the stress-life (S-N) diagram [7]. Consequently, a discernible need materializes to integrate the variability in material properties and the role played by process-related defects into the frameworks of design and structural integrity assessment methodologies, applicable to components and parts produced through additive manufacturing. In preliminary studies [8,9,10], investigations have been conducted to assess the impact of pores induced by wire and arc additive manufacturing on the HCF behavior of Ti-6Al-4V structures. In addition to comprehending the high-cycle fatigue (HCF) behavior, it is equally crucial to delve into the low-cycle fatigue (LCF) behavior for quality assurance purposes. This emphasis is essential because the failure mechanism can undergo changes as plastic strains occur within this region, which pertains to the segment of the S-N diagram encompassing up to approximately $10^{4}$ cycles [7]. While the high-cycle fatigue behavior and its associated influencing factors have seen increasing attention and understanding, the low-cycle fatigue properties of WAAM Ti-6Al-4V components have not yet undergone thorough and comprehensive investigation. However, recent findings suggest that LCF characteristics are contingent on both the manufacturing process and the cyclic deformation process, as evidenced by studies such as [11,12]. Hence, there exists a necessity for comprehensive investigations into the low-cycle fatigue strength and the influencing parameters, such as process-induced defects and microstructure. This paper, in particular, focuses on a detailed examination of these aspects. The analysis is executed through a series of strain-controlled experiments encompassing Ti-6Al-4V specimens manufactured via the WAAM process. To facilitate a comparative assessment of cyclic material behavior against firmly established processes, tests were also conducted utilizing wrought material specimens. Esteemed analysis and assessment models, applicable to low-cycle behavior, were employed for both material conditions, investigating the influence of pores and the microstructure. This was followed by a comprehensive examination of cyclic deformation behavior, and a comparative study was extended to include various other additive manufacturing (AM) processes, namely selective laser melting (SLM), electron beam melting (EBM), and laser engineered net shaping (LENS). The objective of this study can be summarized with the following topics:

- Experimental investigations of the quasi-static and LCF properties of the substrate and WAAM material considering microstructural effects.
- Detailed analysis of the cyclic deformation behavior for both material conditions.
- Evaluation of common LCF model parameters based on the conducted experiments and comparison of the results with other AM processes, such as selective laser melting, electron beam melting and laser engineered net shaping.

The primary scientific contribution of this paper lies in the comprehensive exploration of the influence exerted by process-induced defects and microstructure on both the finite low-cycle fatigue strength and the cyclic deformation behavior of Ti-6Al-4V structures produced through wire arc additive manufacturing. Additionally, the study involves the application of assessment methodologies for cyclic deformation behavior. Notably, the investigation includes an extensive comparative analysis, encompassing wire and arc additive manufacturing (WAAM) in relation to both wrought material and other materials processed through various additive manufacturing methods.

## 2. Materials and Methods

### 2.1. Materials and Additive Manufacturing

In this study, the low-cycle fatigue performance of wire arc additive manufactured Ti-6Al-4V is investigated. Both the conventionally hot-rolled 13 mm thick substrate and the 1.6 mm wire consumable were composed of the grade 5 titanium alloy Ti-6Al-4V. The nominal chemical composition of this alloy, as specified by the manufacturer, can be found in Table 1, which is in accordance to ASTM B348 [13]. In addition, the actual chemical analysis of the manufactured WAAM material, derived from energy dispersive X-ray spectroscopy (EDX), is shown in Table 1. It is comparable to the initial chemical composition of the wire as well as the substrate.

**Figure 1:**
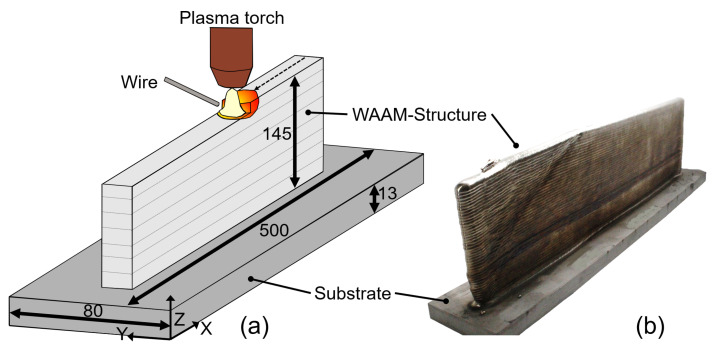
Schematic of the WAAM process (**a**), and WAAM structure used for extraction of test specimen (**b**).

A single WAAM structure was manufactured using a plasma arc welding machine combined with a four-axis gantry robot in order to generate representative material for specimen extraction. During the process, the titanium alloy Ti-6Al-4V was deposited layer-by-layer on a substrate using a 1.6 mm diameter wire consumable, with a double wire feeder for achieving reasonable deposition rates. Figure 1a shows a schematic of the process, with the wire consumable being added layer-by-layer onto the substrate. The process utilized a deposition rate of about 3.3 kg/h, an energy per length of about 10^6^ J/m, an average power of 6 kW, and a travel speed of about 5–6 mm/s. The process parameters used for manufacturing are comparable to data from the literature [16]. To protect the melted weld pool and the heat-affected zone from oxidation and other undesirable contamination, a pure argon-filled box chamber was used. The box chamber is filled with pure argon (99.9999%), and the atmosphere was cleaned by a recirculation device to ensure a clean atmosphere for the process.

In order to extract specimens and characterize the low-cycle fatigue performance, a WAAM structure was built using the previously described process parameters. A single-layer building strategy was utilized, with an alternating deposition direction in each layer, meaning that the deposition direction changed in each layer. Dwell times were set and adopted during the process to ensure a constant interpass temperature of around 400 °C, in accordance with data from literature [17]. One wall was deposited with a total length of about 400 mm on a substrate with overall dimensions of 500 × 80 × 13 mm^3^, as shown in Figure 1b. The effective wall thickness was measured to be about 14 mm. The wall consists of 45 layers with a total height of 145 mm resulting in an average layer height of about 3.2 mm.

### 2.2. Test Procedure

Quasi-static tensile and low-cycle fatigue tests were conducted to investigate the corresponding material behavior of both, substrate and additively manufactured structure. To do so, specimens were extracted from an unprocessed titanium base plate used as WAAM substrate and additionally fabricated specimens from the WAAM structure itself.

For substrate material characterization, specimens were cut in a longitudinal-rolling direction from a plate with approximate dimensions of 210 × 200 × 13 mm^3^. For the WAAM material, specimens were extracted above the sixth deposited layer. This layer was chosen to ensure a similar microstructure and avoid any influence of positioning, assuming a constant heat balance during the process. The specimens in this study were taken from the horizontal, as the first step was to primarily consider the fatigue behavior in the loading direction parallel to the deposition direction.

**Figure 2:**
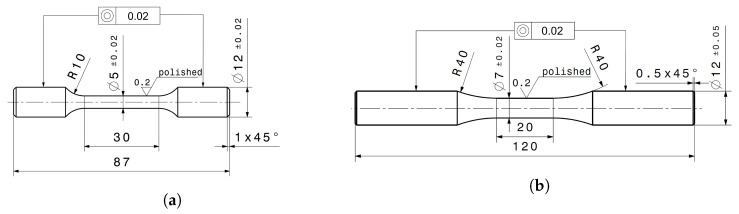
Geometry of test specimen in [mm]. (**a**) Quasi-static tensile specimen; (**b**) Low-cycle fatigue specimen.

To statistically evaluate the mechanical material properties and the LCF performance, a total of 15 fatigue and ten tensile specimens were manufactured out of both the AM structure and the substrate material. The geometry of the tensile test specimens was designed according to ASTM E8 [18]. Round specimens were chosen with a diameter of 5 mm and an initial extensometer length of 25 mm in the test area. A total length of 87 mm and a clamping diameter of 12 mm was utilized, as shown in Figure 2a. The geometry of the round LCF test specimens was defined according to ASTM E606 [19], with an initial extensometer length of 12.5 mm and a diameter of 7 mm in the test area. The geometry for the LCF tests is shown in Figure 2b, with a total length of 120 mm and a diameter of 12 mm in the clamping area. After machining to the specified geometric dimensions, the surface was finished by polishing to avoid any influence of surface roughness and residual stresses from machining on the test results.

**Figure 3:**
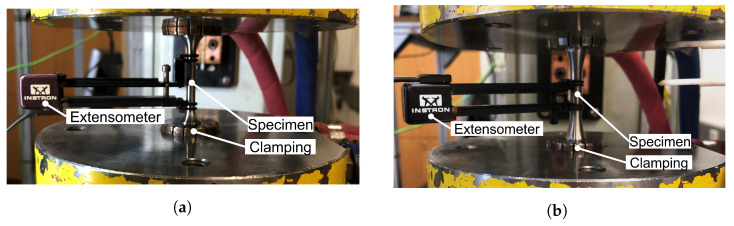
Detailed experimental test setup for material characterization. (**a**) Quasi-static tensile test configuration; (**b**) Low-cycle fatigue test configuration.

A uniaxial, servo-hydraulic test cylinder with a nominal load capacity of 100 kN was used for the experimental investigations, and clamping of specimens was executed with hydraulic grips and clamping sleeves on both sides. Strain during the LCF tests at room temperature (RT) was measured using a contacting Instron extensometer with an initial length of 25 mm for the tensile tests and 12.5 mm for the LCF tests, as shown in Figure 3. Strain was controlled during all tests to ensure a defined and constant strain rate.

As the first step of the experimental investigations, a total of ten tensile tests were conducted to study the quasi-static behavior and to estimate the total strain amplitude levels relevant for the LCF tests. Tensile tests were carried out in a strain-controlled mode until fracture of the specimen, with five specimens taken from the WAAM material and five from the substrate. A strain rate of 2.5 × 10^−3^ s^−1^ was established for all tensile tests according to the standard.

Subsequent LCF tests were conducted under a load strain ratio of $R_{\epsilon }=-1$ with different total strain amplitudes $\varepsilon_{a}$, which were defined based on the previously executed tensile tests. These tests were also performed in a strain-controlled mode until fracture of the specimen, and the total strain amplitudes for the LCF tests were varied between 0.675% and 1.4%. Strain was applied and controlled by a triangular waveform during the tests, in accordance with the standard ASTM E606 [19]. The test frequency was set for each test to ensure a constant strain rate of 1.0 × 10^−2^ s^−1^ for the different total strain amplitude levels. A total of 15 LCF tests were carried out, using seven specimens from the substrate and eight specimens from the WAAM material.

### 2.3. Models for Estimation of the Low-Cycle Fatigue Behavior

To fit the experimental LCF data to a model for the cyclic stress-strain curve, Ramberg–Osgood proposed a relatively simple three-parameter material model [20]. To model the cyclic stress-strain response, the total strain amplitude $\varepsilon_{a}$ is generally split into the elastic $\varepsilon_{a,e}$ and plastic $\varepsilon_{a,p}$ strain amplitudes, as shown in the following Equation (1):

(1) $$\varepsilon_{a}=\varepsilon_{a,e}+\varepsilon_{a,p}$$

The cyclic stress-strain curve is modeled by three parameters in the Ramberg–Osgood model in Equation (2) as follows:

(2) $$\varepsilon_{a}=\frac{\sigma_{a}}{E}+{\left(\frac{\sigma_{a}}{K^{\prime }}\right)}^{\frac{1}{n^{\prime }}}$$

where *E* is the Young’s modulus, $K^{\prime }$ denotes the cyclic hardening coefficient and $n^{\prime }$ is the cyclic hardening exponent. The first addend in Equation (2) represents the elastic part, while the second addend represents the plastic part.

In addition to the previously described Ramberg–Osgood equation, which describes the cyclic deformation behavior, a local strain approach according to Manson–Coffin–Basquin (MCB) is used to relate the total strain amplitude to the number of load cycles to failure [21,22,23]. The total strain amplitude is further divided into the elastic and the plastic parts, whereby the Basquin equation (see Equation (3)) describes the elastic strain and the Manson–Coffin equation (see Equation (4)) describes the plastic strain. Additionally, the elastic and plastic components of the MCB approach are compared to the Ramberg–Osgood equation. As introduced, Equation (3) describes the elastic and Equation (4) the plastic part of the total strain amplitude, with the first term representing the RO model and the second term representing the MCB model:

(3) $$\varepsilon_{a,e}=\frac{\sigma_{a}}{E}=\frac{\sigma_{f}^{\prime }}{E}{\left(2N_{f}\right)}^{b}$$

(4) $$\varepsilon_{a,p}={\left(\frac{\sigma_{a}}{K^{\prime }}\right)}^{\frac{1}{n^{\prime }}}=\varepsilon_{f}^{\prime }{\left(2N_{f}\right)}^{c}$$

where $\sigma_{f}^{\prime }$ is the fatigue strength coefficient, $\varepsilon_{f}^{\prime }$ the fatigue ductility coefficient, and *b* and *c* are the fatigue strength and fatigue ductility exponents. In other words, *b* and *c* represent the slope of the elastic and plastic part of the strain-fatigue diagram, respectively. The final MCB equation in Equation (5) is obtained by combining the elastic and plastic parts, as follows:

(5) $$\varepsilon_{a}=\frac{\sigma_{f}^{\prime }}{E}{\left(2N_{f}\right)}^{b}+\varepsilon_{f}^{\prime }{\left(2N_{f}\right)}^{c}$$

### 2.4. Metallographic and Microstructural Analysis

To investigate the microstructure of the manufactured WAAM material and compare it to the substrate’s condition, one representative cross section sample was cut from the middle at a specific position of X = 250 mm of the WAAM structure in the Y-Z plane (cf. Figure 1b). This metallographic specimen was ground to achieve a flat surface, polished to remove all scratches, and finally etched with Kroll reagent to visualize the microstructure. Following this three-step preparation procedure, the specimen’s surface was documented using a digital optical microscope. For a more detailed characterization of the microstructure, scanning electron microscopy was used.

One fracture surface of each sample was analyzed by mean of a digital optical microscope after the fatigue test runs to investigate the origin of the failure.

## 3. Results

### 3.1. Macro- and Microstructure

**Figure 4:**
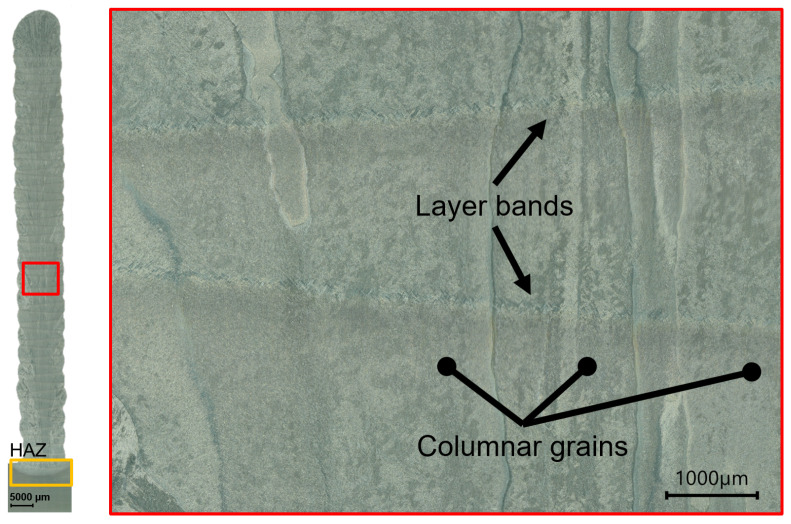
Overview of the extracted specimen and detail of the material’s macrostructure.

A comprehensive depiction of the cross-section is provided in Figure 4, encompassing both a broader perspective and a more intricate examination of the macrostructure of both the WAAM and substrate materials. Notably, this cross-section was extracted from the central region of the WAAM structure as shown in Figure 1. Looking at the overview in Figure 4, a difference in the microstructure between the substrate and the WAAM material can be observed. The substrate material reveals a more refined microstructure, specifically evident in its grain size, when compared to the WAAM structure itself. This contrast is highlighted by the comparison between the yellow rectangle (representing the substrate) and the red rectangle (representing the WAAM material) in the overview of Figure 4. Within the highlighted yellow area in Figure 4, the heat-affected zone (HAZ) is discernible, wherein a gradual grain coarsening is observable from the lower to upper regions. Furthermore, the overview shows a columnar grain structure of the WAAM material, with columnar grains grown perpendicular to the deposition in the Z-direction.

In the detail of the material’s macrostructure in Figure 4, the columnar grains can also be seen in more detail. In general, the columnar grain size in WAAM material is larger than the microstructure of materials manufactured with other additive manufacturing processes, such as selective laser melting [24,25,26]. The columnar grains in WAAM materials span a few millimeters across multiple layers, which can be attributed to a significantly lower temperature gradient during solidification and cooling in comparison to the other processes [27]. This lower solidification velocity is attributed to the higher deposition rates employed in the WAAM process. The higher deposition rates necessitate more power and lead to a wider region of the previous layer being either reheated or even liquefied again. In other words, the large heat input results in a lower temperature gradient. One characteristic of the WAAM macrostructure is the presence of layer bands, as seen in Figure 4. These layer bands are caused by the layer-by-layer manufacturing process and appear similar to a heat-affected zone. The previously deposited layers are repeatedly heated, leading to changes in the microstructure. In the region of these layer bands, the material experiences a temperature below the β transus temperature, resulting in a finer α lamellar transformation structure.

**Figure 5:**
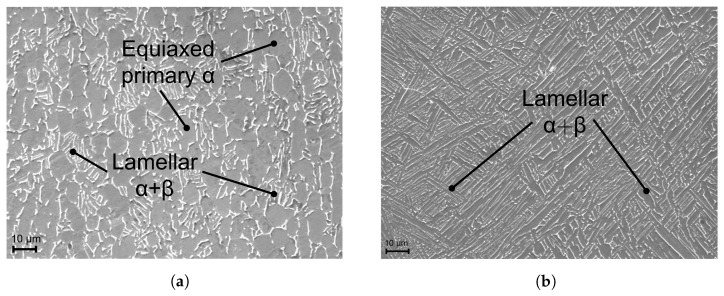
Representative microstructure of the investigated material conditions. (**a**) Substrate material; (**b**) WAAM material.

To compare the microstructure of the substrate with WAAM material, SEM images of different specimen regions were taken. The representative figure of the substrate material (Figure 5a) has been extracted directly from the unprocessed plate, thereby ensuring the absence of any influence stemming from the WAAM process. Furthermore, the representative figure illustrating the WAAM material (Figure 5b) has been sourced from the central region of the WAAM structure, specifically within the red rectangular region delineated in Figure 4, precisely within a columnar grain. Figure 5a shows the SEM image of the substrate, which exhibits a bimodal microstructure consisting of a distribution of equiaxed primary α grains and lamellar α and β phases, whereby these phases are transformed β. In comparison, Figure 5b shows the microstructure of the WAAM material, which consists of a fully lamellar (α+β) microstructure. This fully lamellar microstructure is characteristic for WAAM-manufactured parts, whereas SLM-manufactured Ti-6Al-4V shows a martensitic transformation due to high cooling rates [27].

### 3.2. Quasi-Static Test Results

**Figure 6:**
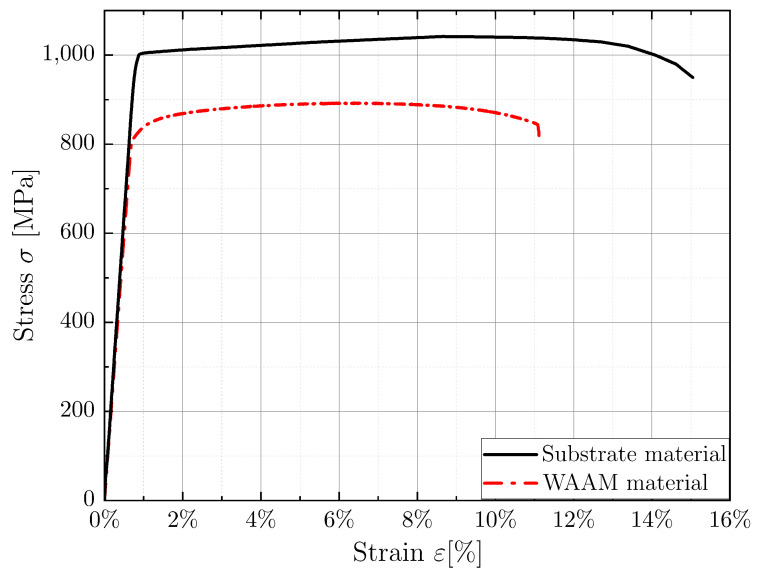
Representative stress-strain tensile test curves for substrate and WAAM material.

Strain-controlled tensile tests were conducted to investigate the elasto-plastic material behavior and determine the initial strain amplitude levels for the LCF tests. As a first step, a comparative analysis was conducted on the stress-strain curves for both material conditions. This process encompassed the presentation of experimentally measured strain data, acquired from the experimental extensometer, in relation to the corresponding measured stress, as elucidated in Section 2. Figure 6 shows two representative stress-strain test curves for the substrate and the WAAM material. When comparing both curves, it is evident that the WAAM material exhibits significantly lower yield strength, ultimate tensile strength, and elongation than the substrate material. However, when comparing the Young’s modulus of both material conditions, it is observed that the slope of the elastic regime and the Young’s modulus of the substrate material are approximately 7% higher than those of the WAAM material.

Experimental data from the conducted quasi-static tensile tests were statistically evaluated and the resulting mechanical properties for both material conditions are presented in Table 2. As previously described, the WAAM material exhibits about −15% lower yield and ultimate tensile strength, a −26% lower elongation, and it is apparent that these material conditions exhibit a slightly higher degree of scatter. These differences in mechanical properties can be attributed to differences in microstructure. Specifically, the substrate exhibits a finer microstructure with smaller grain size than the WAAM material, as shown in Figure 5. In addition, the lower elongation of the WAAM material can be attributed to the presence of internal defects, such as pores, or the influence of a texture in the material [28].

When comparing the experimental results from this study to the minimum requirements for forged and selectively laser melted Ti-6Al-4V as specified in ASTM B381 [29] and ASTM F2924 [30], respectively, it can be seen that all properties meet or exceed the minimum requirements. When compared with the values in the literature of other AM processes, it can be generally observed that SLM [24,31] and cold metal transfer-wire arc additive manufacturing (CMT-WAAM) [8,32] exhibit higher mechanical properties than the WAAM process used in this study. This is primarily due to the finer microstructure resulting from a higher temperature gradient in these processes.

### 3.3. Low-Cycle Fatigue Properties

**Figure 7:**
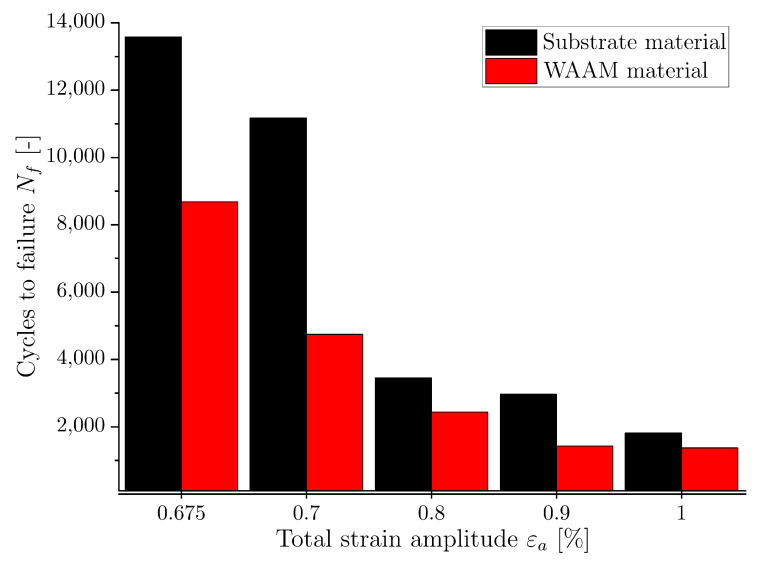
Number of cycles to failure for different amplitudes and material conditions.

In order to give an overview and to compare the LCF behavior, the number of load cycles to failure at different total strain amplitudes was compared and is presented in Figure 7. By comparing the LCF results of the two investigated material conditions, it can be observed that the number of cycles to failure is consistently lower for the WAAM material at all applied strain amplitudes. This is likely due to the presence of larger grains and potential defects, such as pores, in the WAAM material when compared to the substrate material. The presence of pores tends to result in a lower elongation compared to defect-free material and, as a result, in a lower number of load cycles until failure. The difference between the substrate and the WAAM material in terms of cycles to failure becomes in general smaller with increasing amplitudes, see Figure 7.

**Figure 8:**
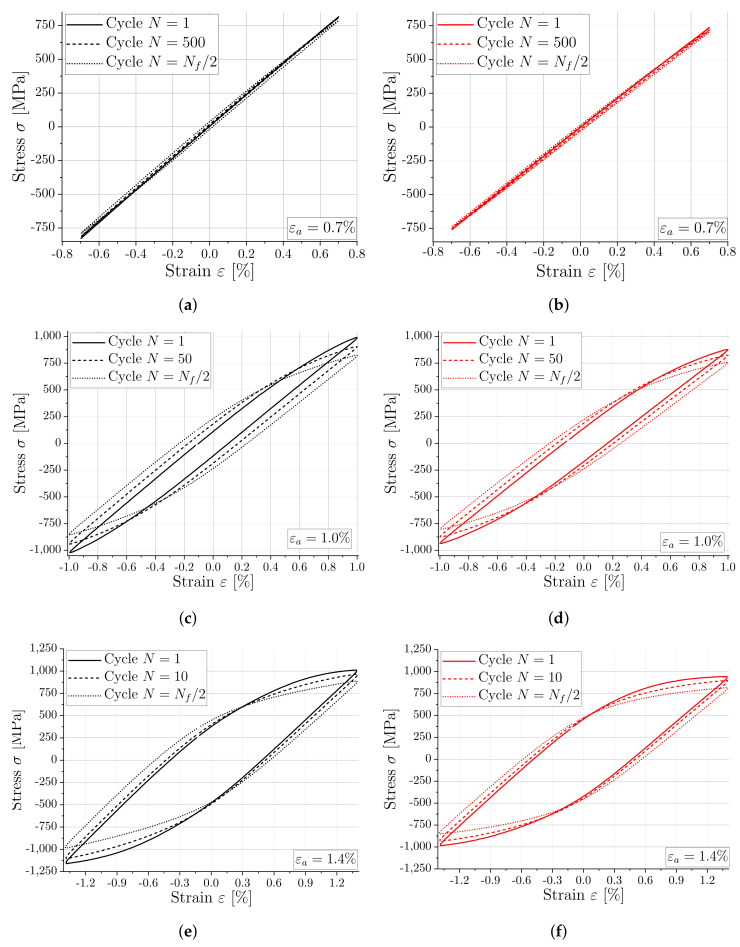
Comparison of representative stress-strain hysteresis at different strain amplitudes and number of load cycles for substrate and WAAM material. (**a**) Substrate material—($\varepsilon_{a}=0.7\%$); (**b**) WAAM material—($\varepsilon_{a}=0.7\%$); (**c**) Substrate material—($\varepsilon_{a}=1.0\%$); (**d**) WAAM material—($\varepsilon_{a}=1.0\%$); (**e**) Substrate material—($\varepsilon_{a}=1.4\%$); (**f**) WAAM material—($\varepsilon_{a}=1.4\%$).

During the experimental LCF tests, specimens were cyclically loaded, and depending on the applied total strain amplitude, plastic strain occurred in addition to the elastic part of the total strain. Due to the characteristic cyclic loading in tension and compression, stress-strain hysteresis could be observed. In this regard, strain measurements were acquired via the extensometer, while stress values were computed by dividing the measured force by the diameter of the specimen, as presented in Section 2.2. Figure 8 presents the cyclic stress-strain hysteresis for both material conditions and selected strain amplitudes. In detail, the cyclic response with total strain amplitudes of 0.7%, 1.0%, and 1.4% for the substrate is depicted in Figure 8a,c,e, while the corresponding response for the WAAM material is shown in Figure 8b,d,f. As materials in general exhibit either cyclic softening or cyclic hardening, it is interesting to investigate whether the stress decreases or increases with each cycle. To study the cyclic behavior regarding softening or hardening, the first and the stabilized cycle at half the number of load cycles to failure are shown in Figure 8. Additionally, the stress-strain response for 500, 50, and 10 load cycles is shown for the related total strain amplitudes of 0.7%, 1.0%, and 1.4%, respectively.

The presented stress-strain curves allow for technical observations to be made by comparing the material conditions and different strain amplitudes. The occurring minimum and maximum stress increases with higher strain amplitudes for both materials, with the WAAM material generally exhibiting lower stresses at a defined strain amplitude compared to the substrate. Furthermore, when the total strain amplitude is low ($\varepsilon_{a}=0.7\%$), the maximum stress lies below the yield strength and no plastic strain occurs, which means that the hysteresis is unincisive, as seen in Figure 8a,b. With an increasing strain amplitude, more plastic strain and an increasing hysteresis can be observed with increasing maximum stress response. Cyclic softening behavior occurs for the investigated material conditions when looking at the stress-strain hysteresis in general.

**Figure 9:**
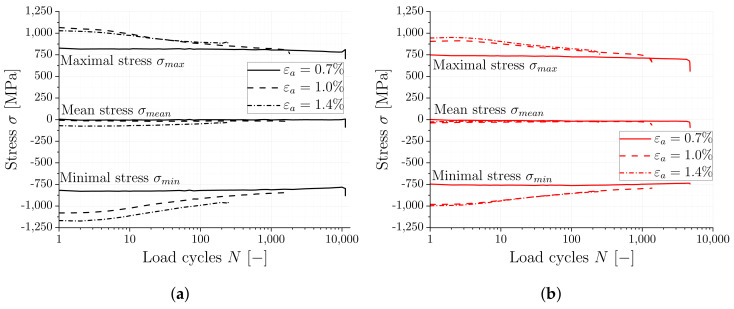
Representative stress over load cycles curves at different strain amplitudes for substrate and WAAM material. (**a**) Substrate material. (**b**) WAAM material.

In order to achieve a more detailed look at the cyclic softening behavior, the evolution of stress for different strain amplitudes over the number of load cycles is presented in Figure 9. According to [12,33], if the ratio between ultimate and yield strength ($UTS/R_{p0.2}$) is below 1.2, the likelihood for the occurrence of cyclic softening is high. The observed ratio for the substrate is 1.05 and for the WAAM material is 1.07, indicating that both investigated materials have a tendency to exhibit cyclic softening under cyclic loading. Softening behavior is indicated by a detailed examination of the evolution of minimal and maximal stress in Figure 9 for different strain amplitudes. No softening is observed for both material conditions for the small strain amplitude of 0.7%. However, for higher strain amplitudes, softening occurs and increases with increasing strain amplitude. Comparing the cyclic softening observed for the two highest strain levels (1.0% and 1.4%), the maximum stress falls in a similar range for both materials. At the same strain amplitudes, the maximum stress for the WAAM material is lower compared to the substrate, as observed in the quasi-static tensile test results.

**Figure 10:**
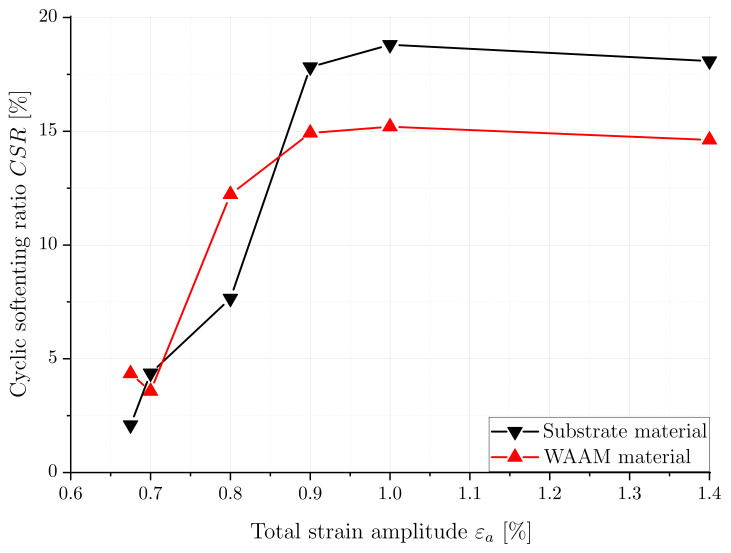
Cyclic softening ratio at different strain amplitudes for substrate and WAAM material.

To quantify the degree of cyclic softening during the LCF tests, the cyclic softening ratio (CSR) can be calculated for both material conditions and different strain amplitudes, as proposed in previous studies such as [12,34,35]. The cyclic softening ratio is calculated using Equation (6):

(6) $$CSR=\frac{\Delta \sigma_{max}-\Delta \sigma_{half}}{\Delta \sigma_{max}}$$

where $\Delta \sigma_{max}$ is the maximum stress range and $\Delta \sigma_{half}$ is the stress range at half of the reached cycles ($N_{f}/2$). The CSR, estimated for all tested strain amplitudes and both material states, is presented in Figure 10. The comparison of softening ratios between the materials shows that only marginal softening occurs below a strain amplitude of 0.75%. However, for strain amplitudes above 0.8%, the softening ratio increases to 15% for the WAAM and 18% for the substrate and reaches a nearly constant value. It is evident that the softening of the substrate material occurs at a later stage compared to that of the WAAM material. Additionally, the substrate exhibits higher cyclic softening compared to the WAAM material, which can be attributed to its higher maximum stress.

**Figure 11:**
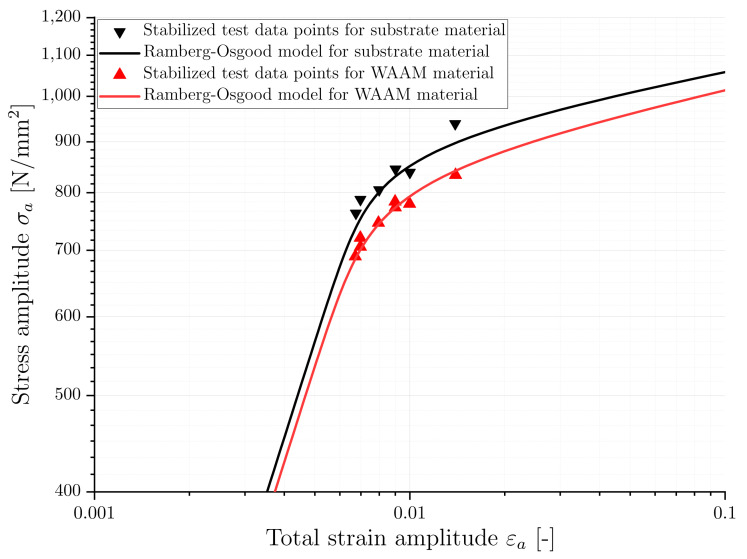
Cyclic deformation behaviour of substrate and WAAM material.

In contrast to the stress-strain response observed in monotonic tensile tests, cyclic loading can result in either hardening or softening, as previously mentioned. The cyclic stress-strain curve provides this additional cyclic information. It is determined by the stabilized hysteresis at half of the load cycles to failure ($N_{f}/2$) of LCF tests at different strain amplitudes. The experimental data of both material conditions are fitted to the previously described Ramberg–Osgood model, and the determined model parameters are presented in Table 3. In addition, Figure 11 shows the experimentally determined, stabilized data points for different strain amplitudes and the fitted Ramberg–Osgood stress-strain curves for both material conditions. It can be observed that the substrate exhibits higher values for the corresponding stress amplitude compared to the WAAM material. Specifically, the cyclic hardening coefficient is higher for the substrate while the hardening exponent is slightly smaller.

**Figure 12:**
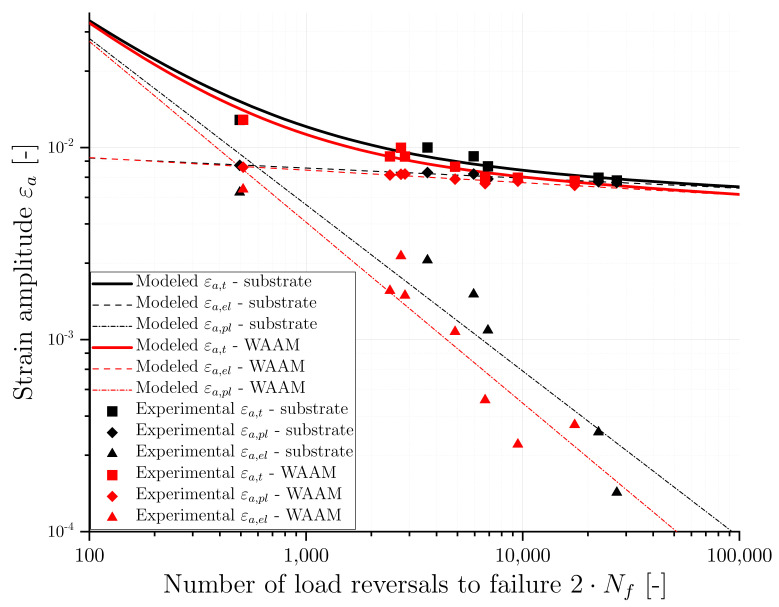
Experimental and modeled strain-life fatigue behaviour of substrate and WAAM material.

The parameters of the previously described MCB equation involves four parameters ($\sigma_{f}^{\prime },\varepsilon_{f}^{\prime },b,c$), which were determined using the least-squares fitting method by fitting the elastic and plastic strain components measured directly from the experimental LCF data. Table 4 presents the determined MCB parameters, and Figure 12 shows the LCF test results for the model fit and the modeled MCB curves. Towards the relationship of total strain and cycles to failure, the elastic and plastic parts are separately shown by straight lines in the figure. From Figure 12, it can be observed that there is a small gap between the two material conditions for the total strain life curves. The gap between the conditions is increasing with an increasing number of load cycles, corresponding to higher plasticity in the WAAM material. The substrate material has a slightly higher transition point than the WAAM material, implying that plastic deformation has a more significant impact on the fatigue life of the WAAM material than the substrate. This phenomenon could arise from the presence of defects or disparities in the microstructure.

### 3.4. Fractography

**Figure 13:**
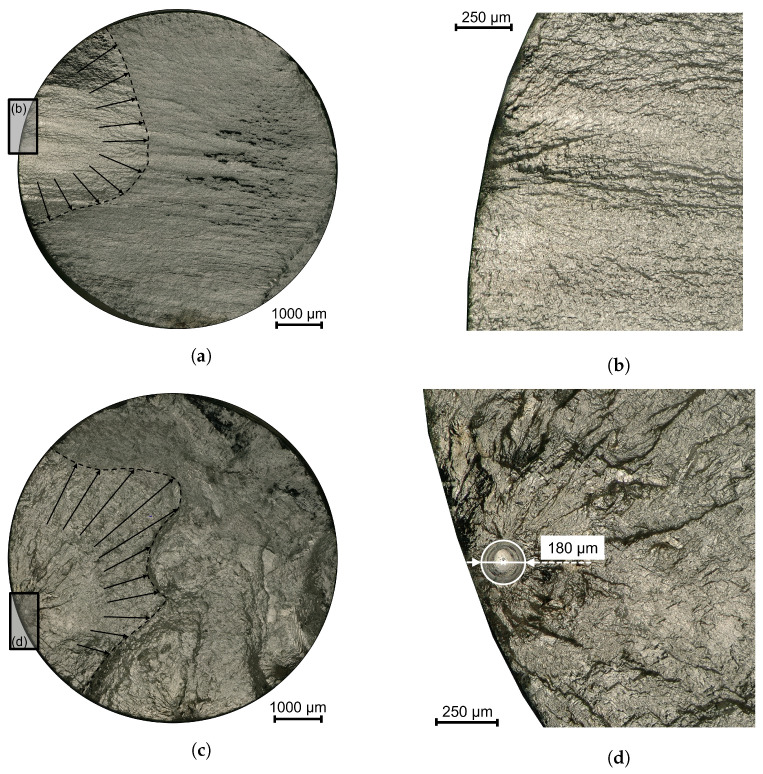
Representative overview of the fracture surface and detail on the crack initiation for both material conditions. (**a**) Substrate material—Overview fracture; (**b**) Substrate material—Detail crack initiation; (**c**) WAAM material—Overview fracture; (**d**) WAAM material—Detail crack initiation.

After conducting the LCF tests and failure of the specimens, the fractured surfaces of each specimen were analyzed to investigate the fatigue crack initiation for both material conditions. Figure 13 provides a representative overview of two fracture surfaces: one for the substrate (Figure 13a) and the other for the WAAM material (Figure 13c). The figure also includes detailed images of the crack initiation sites, which are presented in Figure 13b for the substrate and Figure 13d for the WAAM material. Fatigue crack initiation was observed in all substrate specimens due to microstructural inhomogeneities, while in the WAAM material, all crack initiations occurred at or next to pores near the surface. In addition to the detailed and marked crack initiation sites, the zone of crack propagation and final fracture are clearly visible, with the size of the crack propagation zone decreasing as the strain amplitude increases, indicating faster crack propagation.

## 4. Discussion

### 4.1. LCF Comparison across Different AM Processes

The microstructure of Ti-6Al-4V structures produced through different AM processes strongly influences their LCF properties. Collections of data from the literature on various AM processes were used to compare and summarize the fatigue properties of the material investigated in this study with previously reported data. This study investigates the LCF properties of a plasma-based WAAM process and its substrate material as a reference. To perform a direct comparison with the same AM process, data from [11], which was also produced via plasma WAAM, was used. Additionally, four data sets from the literature were used, which are related to powder-based processes using selective laser melting [24,36], electron beam melting [37], and laser engineered net shaping [38]. In addition, the LCF properties were compared to those of the substrate material to highlight the general influence of AM processes.

**Figure 14:**
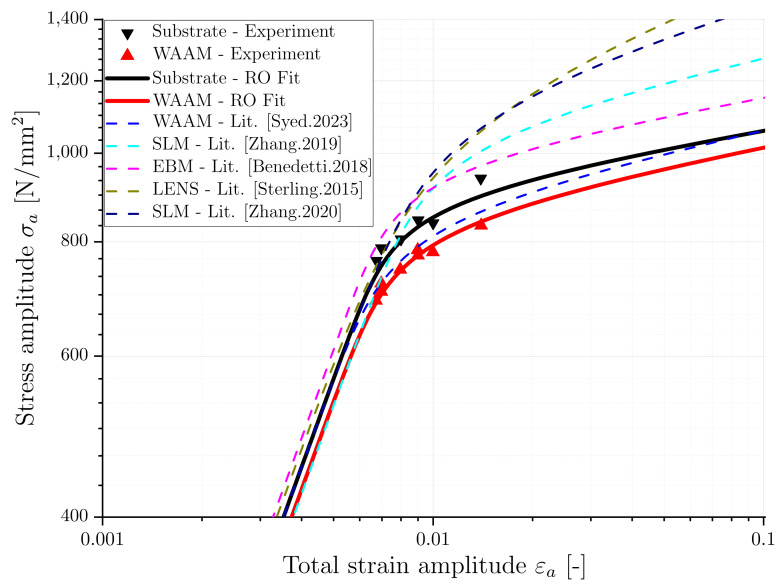
Comparison of cyclic deformation behavior for different AM processes [11,24,36,37,38].

Comparing the cyclic deformation behavior across all datasets was the first step. The Ramberg–Osgood parameters identified in this study, as well as those from the literature, are presented in Table 5. To enable direct comparison of the resulting cyclic deformation behavior in terms of the stress-strain relationship, strain amplitude was calculated for two specified stress amplitudes using Equation (2), whereby the results are added and presented in Table 5. Furthermore, Figure 14 compares the fitted stress-strain curves from this study with the resulting curves from data from the literature, as described in Table 5.

Several observations can be made when comparing the resulting stress-strain curves for the different AM processes and the substrate. Initially, up to a strain amplitude of approximately $\varepsilon_{a}$ = 0.65%, no significant differences between the curves can be observed. For a specified stress of $\sigma_{a}$ = 600 MPa, the estimated strain amplitude shows no significant difference for the different AM processes. This is due to the fact that in this region, no major plastic strain occurs, and linear material behavior can be assumed. Moreover, the Young’s modulus, which describes the linear region, is found to be in the same range for all datasets. Looking at the region with higher strains, the difference in cyclic deformation behavior increases due to the increasing influence of plasticity and different microstructure. The material produced through plasma-based WAAM, as reported in the literature [11], shows behavior comparable to the materials in this study. However, the materials produced through SLM [24,36], EBM [24], and LENS [38] processes exhibit higher plasticity than the WAAM and substrate material, owing to the development of a finer microstructure due to higher cooling rates within these processes. When considering a specified stress of $\sigma_{a}$ = 1000 MPa, the resulting strain amplitude exhibits a noteworthy difference among the various AM processes. While the materials produced via SLM, EBM, and LENS show a comparable mean amplitude of 1.3%, the WAAM material from this study exhibits an amplitude of 8.3%, which is approximately six times higher.

**Figure 15:**
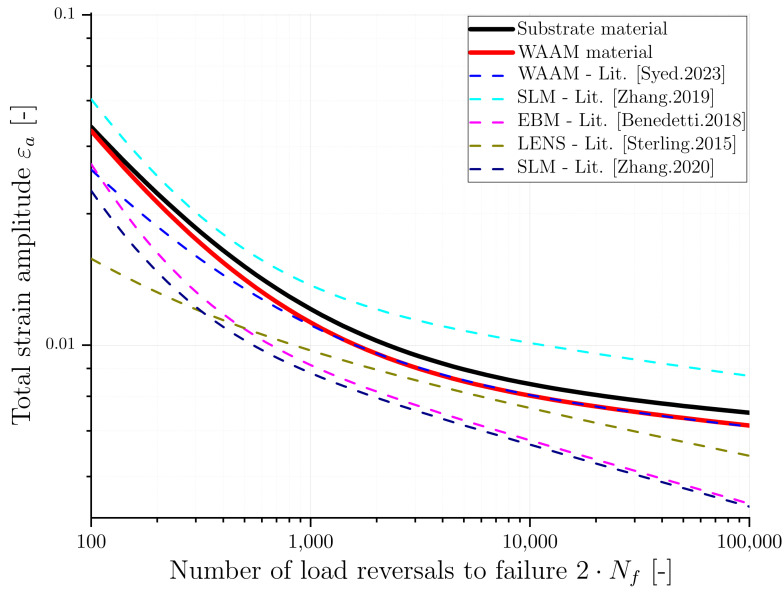
Comparison of strain fatigue life curves for different AM processes [11,24,36,37,38].

As a second step, the strain-life curves of this study’s materials and the fitted MCB parameters were compared to data from the previously introduced literature for different AM processes. In Table 6, the MCB parameters are presented, and the strain amplitude was estimated for two specified numbers of load cycles using Equation (5) to compare the obtained values. These values for the load cycles are also added to Table 6 for reference. The strain-life curves for different AM processes based on the fitted and MCB parameters in the literature are compared and plotted in Figure 15. The behavior of the WAAM material from this study is compared with the same process from the literature [11], and no significant difference is found until *N* = 1000. However, for total strain amplitudes larger than 0.01, the WAAM material from this study exhibits a higher fatigue life. In contrast, the EBM [37], LENS [38], and SLM [36] materials exhibit mean −24%, −34%, and −32% lower strain amplitudes at the same number of load reveals to failure, respectively, compared to the WAAM material. One possible reason is the reported presence of defects and inhomogeneities in their material [36,37,38]. Contrariwise, the SLM [24] material demonstrates a mean of 34% higher strain amplitudes. Lastly, a comparison of the substrate with the WAAM material reveals a mean of 6% higher strain amplitudes for the substrate.

### 4.2. Accuracy of Simplified Manson–Coffin–Basquin Estimation Models

It is noted that the MCB equation is dependent on four parameters, which can be obtained through fitting experimental data to Equation (5). However, to simplify the process, the literature provides estimation models for these parameters [39,40]. In [39], a Modified Universal Slope Equation (MUSE) is proposed for all materials based on 47 investigated materials, where the slopes (exponents) for the elastic (*b* = −0.09) and plastic (*c* = −0.56) parts are universalized. The fatigue strength coefficient $\sigma_{f}^{\prime }$ and fatigue ductility coefficient $\varepsilon_{f}^{\prime }$ are estimated by correlations of the Young’s modulus *E*, the ultimate tensile strength UTS, and the elongation *A*. The final estimation model for the MCB equation is presented in Equation (7):

(7) $$\varepsilon_{a,est,M1}=0.623{\left(\frac{UTS}{E}\right)}^{0.832}{\left(2N_{f}\right)}^{-0.09}+0.0196A^{0.155}{\left(\frac{UTS}{E}\right)}^{-0.53}{\left(2N_{f}\right)}^{-0.56}$$

In addition to the first simplified approach, [40] proposed an even more unified approach for the estimation of the MCB parameters. This approach is called the “universal material law” (UML) [40], where the elastic (*b* = −0.095) and plastic (*c* = −0.69) slopes (exponents) and the fatigue ductility exponent ($\varepsilon_{f}^{\prime }$ = 0.35) are constant and the fatigue strength coefficient is dependent on the ultimate tensile strength and the Young’s modulus. The final estimation model for the MCB approach according to [40] is shown in Equation (8):

(8) $$\varepsilon_{a,est,M2}=1.67\left(\frac{UTS}{E}\right){\left(2N_{f}\right)}^{-0.095}+0.35{\left(2N_{f}\right)}^{-0.69}$$

**Figure 16:**
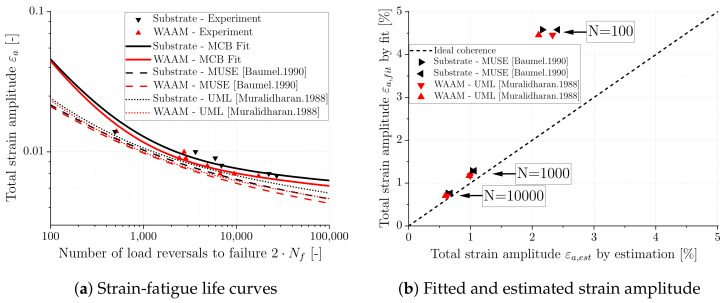
Strain-fatigue life curves and comparison of fitted and modeled stress-strain [39,40].

In order to evaluate the accuracy of the simplification models for the MCB equation, the strain-fatigue life curves are calculated for the two models and plotted in Figure 16a. Furthermore, the fitted fatigue curves for the substrate and the WAAM material are shown in Figure 16a. To clarify the accuracy of estimation, the fitted strain amplitude $\varepsilon_{a,fit}$ is plotted over the estimated strain amplitude $\varepsilon_{a,est}$ in Figure 16b. Comparing the estimated curves with the fitted data, it can be seen that both models tend to produce conservative and underestimated curves, resulting in lower fatigue life predictions for both the substrate and the WAAM material. The model proposed by [39] yields, on average, a −29% error for the substrate and a −27% error for the WAAM material, while the model by [40] shows a −24% and −23% error, respectively. The error between the estimation and the fit is generally smaller for higher numbers of load cycles (*N* = 10,000), and increases with higher strain amplitudes. Overall, the “universal material law” proposed by [40] appears to provide better and more acceptable results, especially considering the simplicity of the approach.

## 5. Conclusions

Based on the experimental investigations and further analysis of the quasi-static tension, cyclic deformation, and strain-fatigue life behavior of the Ti-6Al-4V substrate and WAAM material presented in this paper, the following scientific conclusions can be drawn:

- The microstructure of the WAAM material was found to be different from the substrate material, with a coarser and more heterogeneous grain structure, as well as an increased amount of defects. These differences in microstructure can explain the observed differences in mechanical behavior and low-cycle fatigue performance between the two material conditions. Specifically, the presence of defects in the WAAM material may act as stress concentrators and reduce its fatigue life compared to the substrate material.
- The tensile test results showed that the WAAM material has about −15% lower ultimate tensile strength and a smaller elongation at fracture compared to the substrate material. These differences can be explained with the differences in microstructure and the presence of defects. The presence of defects reduces the elongation and a coarser microstructure reduces the tensile strength, as observed within the WAAM material. These findings emphasize the importance of understanding the microstructure and its effect on the mechanical properties of WAAM materials, as well as the need for further research to optimize the manufacturing process and improve the properties of WAAM materials for various applications.
- Experimental, strain-controlled LCF tests were conducted to study the cyclic deformation behavior and the strain-fatigue life of WAAM and substrate material. Thereby, the LCF tests reveal in general a lower fatigue strength of the WAAM material compared to the substrate. In contrast to the substrate, fractographic analysis showed that crack initiation in the WAAM material occurred exclusively at pores within the specimen rather than at microstructural features.
- The experimental results showed that both the substrate and WAAM materials exhibited the typical cyclic softening behavior observed in Ti-6Al-4V, which became more pronounced at higher strain amplitudes. The cyclic deformation behavior of the WAAM material was found to be different from the substrate material, with larger hysteresis loops and with an average −6% lower cyclic softening rate.
- Experimental data were used to fit the Ramberg–Osgood model for cyclic deformation behavior and the Manson–Coffin–Basquin model for strain-life curves. The fitted models were then used to compare the cyclic deformation behavior and strain-life performance of the WAAM and substrate materials with other AM processes.
- Comparisons with data from the literature on materials produced using other AM processes, such as SLM, EBM, and LENS, reveal that the presence of more localized heat sources and the use of powders lead to finer microstructures and about 13% higher quasi-static mechanical properties (e.g., yield strength). However, these AM processes also tend to produce more defects, resulting in overall −17% lower fatigue life compared to WAAM material.
- Two simplified approaches for the estimation of the MCB parameters were evaluated and compared to the fitted experimental data. Both models showed an underestimation (28% for the MUSE and about 23% for the UML) and conservative curves resulting in lower fatigue life for both the substrate and the WAAM material.

Further research is needed to better understand the behavior of WAAM materials under different loading conditions, and to optimize the manufacturing process to minimize defects and improve the mechanical properties of the final product. This could include investigating the effect of varying distinct process parameters (such as interpass temperature, heat input, etc.), different post-processing treatments and material compositions on the mechanical behavior of WAAM materials. Additional effects on the fatigue strength of structures will be investigated in future research, including the impact of residual stresses on the fatigue life and the influence of anisotropy resulting from directional solidification during additive manufacturing.

## Figures and Tables

**Table 1:** Chemical composition of substrate, wire and WAAM material in weight—%.

	Source	Al	V	Fe	O	N	C	H	Ti
Substrate	[14]	5.50–6.75	3.50–4.50	<0.30	<0.20	<0.05	<0.08	<0.015	Rest
Wire	[15]	6.00	4.00	<0.15	0.18	<0.03	<0.05	<0.01	
WAAM	EDX	5.96 ± 0.13	4.28 ± 0.17	0.14 ± 0.04	-	-	-	-	

**Table 2:** Statistical evaluated quasi-static tensile test results.

Material	Young’s Modulus	Yield Strength	Tensile Strength	Elongation
	E [GPa]	$\sigma_{y,0.2}$ [MPa]	UTS [MPa]	A [%]
Substrate	119 ± 3	1001 ± 14	1054 ± 16	15 ± 2
WAAM material	111 ± 3	838 ± 20	896 ± 22	11 ± 2

**Table 3:** Ramberg–Osgood parameters for the cyclic stress-strain curve.

Material	Cyclic Hardening	Cyclic Hardening
	Coefficient $K^{\prime }$ [MPa]	Exponent $n^{\prime }$ [-]
Substrate	1223	0.061
WAAM material	1197	0.069

**Table 4:** Manson–Coffin–Basquin parameters for the strain-fatigue life curve.

Material	Fat. Strength	Fat. Ductility	Fat. Strength	Fat. Ductility
	Coefficient $\sigma_{f}^{\prime }$ [MPa]	Coefficient $\varepsilon_{f}^{\prime }$ [-]	Exponent b [-]	Exponent c [-]
Substrate	1275	1.99	−0.052	−0.866
WAAM material	1283	2.73	−0.065	−0.942

**Table 5:** Comparison of Ramberg–Osgood parameters for different AM processes.

Data	Process	$K^{\prime }$ [MPa]	$n^{\prime }$ [-]	$\varepsilon_{a}$ [%] for $\sigma_{a}$ = [MPa]	
				600 MPa	1000 MPa
This study	Substrate	1223	0.061	0.51	4.43
	WAAM	1197	0.069	0.54	8.25
[11]	WAAM	1273	0.077	0.53	5.20
[24]	SLM	1535	0.078	0.57	1.36
[37]	EBM	1337	0.063	0.49	1.81
[38]	LENS	2145	0.134	0.51	1.18
[36]	SLM	1875	0.105	0.53	1.14

**Table 6:** Comparison of Manson–Coffin–Basquin parameters for different AM processes.

Data	Process	$\sigma_{f}^{\prime }$ [MPa]	$\varepsilon_{f}^{\prime }$ [-]	*b* [-]	*c* [-]	$\varepsilon_{a}$ [%] for	
						N = 100	N = 10,000
This study	Substrate	1275	1.99	−0.052	−0.866	4.6	0.76
	WAAM	1283	2.73	−0.065	−0.942	4.5	0.70
[11]	WAAM	1270	0.97	−0.061	−0.791	3.4	0.71
[24]	SLM	2452	7.78	−0.092	−1.142	5.6	1.02
[37]	EBM	3600	21.00	−0.190	−1.480	3.5	0.51
[38]	LENS	2618	0.26	−0.136	−0.797	1.8	0.65
[36]	SLM	3119	15.35	−0.186	−1.470	2.9	0.50

## Data Availability

Not applicable.

## Abbreviations

The following abbreviations are used in this manuscript:

AM	Additive manufacturing
CSR	Cyclic softening ratio
EBM	Electron beam melting
EDX	Energy dispersive X-ray spectroscopy
HAZ	Heat-affected zone
HCF	High-cycle fatigue
LCF	Low-cycle fatigue
LENS	Laser engineered net shaping
MCB	Manson–Coffin–Basquin
MUSE	Modified Universal Slope Equation
RO	Ramberg–Osgood
RT	Room temperature
SEM	Scanning electron microscopy
SLM	Selective laser melting
UML	Universal material law
WAAM	Wire arc additive manufacturing
*A*	Elongation at fracture
*b*	Fatigue strength exponent
*c*	Fatigue ductility exponent
$\Delta \sigma_{max}$	Maximum stress range
$\Delta \sigma_{half}$	Stress range at half load cycles
*E*	Young’s modulus
$K^{\prime }$	Cyclic hardening coefficient
$n^{\prime }$	Cyclic hardening exponent
$N_{f}$	Number of load cycles until failure
$R_{\epsilon }$	Load strain ratio
UTS	Tensile strength
$\sigma_{y,0.2}$	Yield strength
$\sigma_{a}$	Stress amplitude
$\sigma_{f}^{\prime }$	Fatigue strength coefficient
$\varepsilon_{a}$	Total strain amplitude
$\varepsilon_{a,e}$	Elastic strain amplitude
$\varepsilon_{a,p}$	Plastic strain amplitude
$\varepsilon_{f}^{\prime }$	Fatigue ductility coefficient
